# The relationship between myocardial fibrosis and myocardial microRNAs in dilated cardiomyopathy: A link between mir‐133a and cardiovascular events

**DOI:** 10.1111/jcmm.13535

**Published:** 2018-01-29

**Authors:** Paweł Rubiś, Justyna Totoń‐Żurańska, Sylwia Wiśniowska‐Śmiałek, Ewa Dziewięcka, Maria Kołton‐Wróż, Paweł Wołkow, Ewelina Pitera, Lucyna Rudnicka‐Sosin, Ann C. Garlitski, Andrzej Gackowski, Piotr Podolec

**Affiliations:** ^1^ Department of Cardiac and Vascular Diseases John Paul II Hospital Krakow Poland; ^2^ Center for Medical Genomics OMICRON Jagiellonian University Medical College Krakow Poland; ^3^ Department of Pathology John Paul II Hospital Krakow Poland; ^4^ Tufts Medical Center Boston MA USA; ^5^ Jagiellonian University Medical Collage Krakow Poland

**Keywords:** biopsy, dilated cardiomyopathy, fibrosis, microRNA, prognosis

## Abstract

It is unknown whether fibrosis‐associated microRNAs: miR‐21, miR‐26, miR‐29, miR‐30 and miR‐133a are linked to cardiovascular (CV) outcome. The study evaluated the levels of extracellular matrix (ECM) fibrosis and the prevalence of particular microRNAs in patients with dilated cardiomyopathy (DCM) to investigate any correlation with CV events. Methods: Seventy DCM patients (48 ± 12 years, EF 24.4 ± 7.4%) underwent right ventricular biopsy. The control group was comprised of 7 patients with CAD who underwent CABG and intraoperative biopsy. MicroRNAs were measured in blood and myocardial tissue via qPCR. The end‐point was a combination of CV death and urgent HF hospitalization at the end of 12 months. There were differential levels of circulating and myocardial miR‐26 and miR‐29 as well as myocardial miR‐133a when the DCM and CABG groups were compared. Corresponding circulating and myocardial microRNAs did not correlate with one another. There was no correlation between microRNA and ECM fibrosis. By the end of the 12‐month period of the study, CV death had occurred in 6 patients, and a further 19 patients required urgent HF hospitalization. None of the circulating microRNAs was a predictor of the combined end‐point; however, myocardial miR‐133a was an independent predictor in unadjusted models (HR 1.53; 95% CI 1.14‐2.05; *P* < .004) and adjusted models (HR 1.57; 95% CI 1.14‐2.17; *P* < .005). The best cut‐off value for the miR‐133a level for the prediction of the combined end‐point was 0.74 ΔCq, with an AUC of 0.67. The absence of a correlation between the corresponding circulating and myocardial microRNAs calls into question their cellular source. This study sheds new light on the role of microRNAs in ECM fibrosis in DCM, which warrants further exploration.

## 1 INTRODUCTION

Fibrosis of extracellular matrix (ECM) is a major pathology contributing to the progression from compensated dilated cardiomyopathy (DCM) to heart failure (HF).^1^ Increasing evidence indicates that microRNAs are important factors in cardiac remodelling, including ECM fibrosis. Thus far, five microRNAs, namely miR‐21, miR‐26, miR‐29, miR‐30 and miR‐133a, have been found to be implicated in ECM fibrosis, mostly in animal studies.^2^, ^3^, ^4^ MicroRNAs function primarily inside cells and tissues, e.g. the myocardium; however, microRNAs have been recently detected in blood samples.^5^, ^6^ We examined: (i) the relationship between circulating and myocardial microRNAs, (ii) the relationship between myocardial microRNAs and ECM fibrosis, and (iii) the associations between microRNAs and cardiovascular (CV) outcome.

## 2 MATERIALS AND METHODS

### 2.1 Study population

Over a period of 14 months, 70 consecutive DCM patients were enrolled. DCM was diagnosed in line with currently accepted criteria.^7^, ^8^ The study complies with the World Medical Association Declaration of Helsinki, and the study protocol was approved by the institutional review board and the ethics committee. All patients gave written informed consent.

The control group was comprised of 7 patients with CAD who underwent CABG. During open‐heart surgery, transmural needle biopsies of the anterior LV wall were performed.^2^

### 2.2 Endomyocardial biopsy

Biopsy was performed in accordance with typical guidelines.^9^ Fibrosis was determined by a pathologist blinded to the clinical data. Collagen volume fraction (CVF) was assessed by quantitative morphometry.^10^

### 2.3 Laboratory measurements

RNA was extracted from 100 μL of plasma performed with a mirVana kit (Life Technologies). Two microlitres of extracted RNA was used to perform reverse transcription with a TaqMan Advanced MicroRNA cDNA Synthesis Kit (Life Technologies). qPCR was conducted on 384‐well plates with TaqMan Advanced Master Mix and TaqMan Advanced Assays targeting: hsa‐miR‐21‐5p, hsa‐miR‐29b‐3p, hsa‐miR‐30c‐5p, hsa‐miR‐133a‐3p, hsa‐mir‐26a‐5p and hsa‐miR‐423‐3p.^11^ Fifteen microlitre reactions were prepared with pipetting station Bravo (Agilent Technologies), and a real‐time reaction was run and read on CFX384 Real‐Time PCR Detection System (Bio‐Rad).

### 2.4 Statistical analysis

The combined end‐point was composed of CV death and urgent HF hospitalization by the 12‐month point. To examine any correlation between microRNAs and end‐points unadjusted and adjusted for age, duration of disease, CVF, EF and NT‐proBNP, Cox proportional hazard analyses were performed.

## 3 RESULTS

### 3.1 Baseline characteristics

At the 12‐month follow‐up, CV death had occurred in 6 (8.6%) patients and urgent HF hospitalization had been required for 19 (27.1%) patients. Thus, the combined end‐point occurred in 25 patients (group 1), whereas the remaining 45 patients (group 2) were free from events. Patients with event‐free survival had a significantly shorter duration of disease (17.8 ± 28.2 vs 36 ± 44.3 months, *P* < .05) and smaller LV end‐diastolic diameters (33.8 ± 5.4 vs 38.8 ± 8.4 mm/m^2^; *P* < .004); however, LV volumes and EF were similar between the two groups. Patients from group 2 had less pulmonary hypertension [11 (24.4%) vs 16 (66.7%); *P* < .001], higher peak oxygen uptake (18.3 ± 6.1 vs 14.5 ± 5.5 mL/kg per min; *P* < .05) and lower NT‐proBNP (2426.8 ± 4247 vs 5069.7 ± 6852 pg/mL; *P* < .05).

### 3.2 Comparison of microRNAs between DCM and CABG groups

DCM patients were younger (48.04 ± 12.1 vs 72.5 ± 3.4 years; *P* < .05) and had lower EF (24.4 ± 7.4 vs 55.6 ± 3.6%; *P* < .001). Both circulating miR‐26 (−0.47 ± 0.79 vs 0.71 ± 0.75 ΔCq; *P* < .05) and tissue miR‐26 (−1.4 ± 0.77 vs 1.9 ± 0.12 ΔCq; *P* < .05) were down‐regulated in DCM, whereas both circulating miR‐29 (7.71 ± 2.78 vs 3.8 ± 1.2 ΔCq; *P* < .01) and myocardial miR‐29 (1.2 ± 0.9 vs 0.02 ± 0.18 ΔCq; *P* < .01) were up‐regulated in DCM. Circulating miR‐133a was similar in both groups, but myocardial miR‐133a (0.91 ± 0.97 vs 1.93 ± 0.11 ΔCq; *P* < .05) was down‐regulated in DCM.

### 3.3 The relationship between the corresponding circulating and myocardial microRNAs

Only miR‐21 was more highly expressed in the myocardium (1.08 ± 0.97 ΔCq) compared to blood levels (0.14 ± 0.6 ΔCq). The remaining microRNAs were at much higher concentrations in the bloodstream compared to cardiac tissue: miR‐26 (blood: −0.02 ± 0.79 vs myocardium: −1.41 ± 0.77 ΔCq; *P* < .001), miR‐29 (blood: 2.78 ± 0.78 vs myocardium: 1.22 ± 0.9 ΔCq; *P* < .001), miR‐30 (blood: 4.39 ± 1.21 vs myocardium: −0.42 ± 1.43 ΔCq; *P* < .001) and miR‐133a (blood: 7.58 ± 2.28 vs myocardium: 0.91 ± 0.97 ΔCq; *P* < .001). None of the corresponding microRNAs correlated with one another in terms of their blood vs myocardium measurements.

### 3.4 MicroRNAs, fibrosis and CV outcome

**Figure 1:**
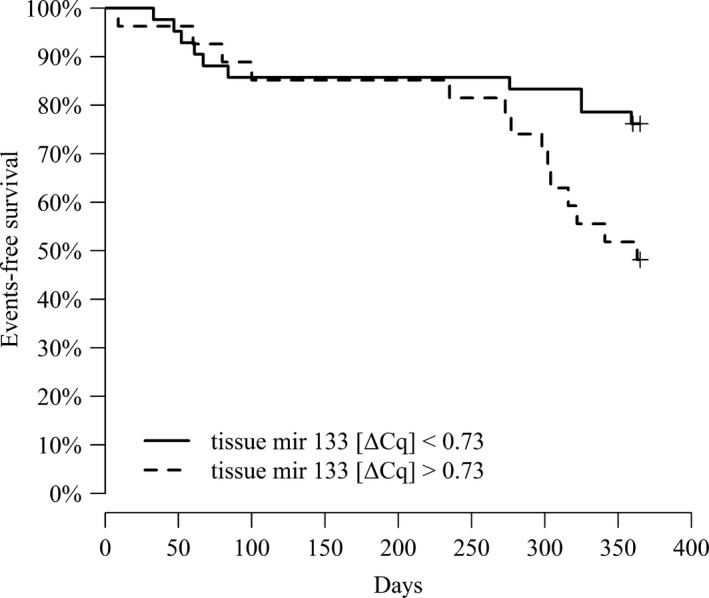
Kaplan–Meier 12‐month survival curve for miR‐133a with cut‐off value of 0.73 [ΔCq]. No difference in the combined end‐point was found between the groups stratified by miR‐133a [ΔCq] value derived from the ROC analysis. The event curves are initially superimposed but begin to diverge after approximately 200 days

No correlation was observed between myocardial microRNAs and fibrosis. There was no difference in any of the circulating microRNAs between patients with or without an event. However, tissue miR‐133a was more highly expressed in patients with an event (Table 1). None of the circulating microRNAs was a predictor of the combined end‐point. Among myocardial microRNAs, myocardial miR‐133a was the only predictor of the end‐point in unadjusted (HR 1.53; 95% CI 1.14‐2.05; *P* < .004) and adjusted (HR 1.57; 95% CI 1.14‐2.17; *P* < .005) models. The best cut‐off value for miR‐133a level for the prediction of end‐point was 0.73 ΔCq, with a sensitivity of 58.3% and specificity of 71.1% (AUC 0.666). Patients with myocardial miR‐133a <0.73 ΔCq had significantly more CV events (HR 2.49; 95% CI 1.1‐5.6; *P* = .03) than those with miR‐133a ≥0.73 ΔCq (see Figure 1).

**Table 1:** Comparison of circulating and myocardial microRNAs between patients with and without a combined endpoint

Parameter	Group 1 (n = 45)	Group 2 (n = 25)	*P*‐value
mir21 [ΔCq]	0.08 ± 0.61	0.23 ± 0.57	.33
mir26 [ΔCq]	−0.17 ± 0.8	0.18 ± 0.75	.08
mir29 [ΔCq]	2.77 ± 0.53	2.8 ± 1.09	.89
mir‐30 [ΔCq]	4.16 ± 1.03	4.57 ± 1.4	.17
mir133a [ΔCq]	7.6 ± 2.1	7.4 ± 2.7	.69
tissue mir21 [ΔCq]	1.13 ± 0.96	1.01 ± 0.99	.58
tissue mir26 [ΔCq]	−1.44 ± 0.82	−1.34 ± 0.67	.61
tissue mir29 [ΔCq]	1.31 ± 0.92	1.06 ± 0.85	.29
tissue mir‐30 [ΔCq]	−0.36 ± 1.7	−0.51 ± 0.66	.33
tissue mir133a [ΔCq]	0.65 ± 0.75	1.36 ± 1.15	.01

Data are presented as mean ± SD.

## 4 DISCUSSION

The biological role and cellular source of circulating microRNAs are largely unknown.^12^ Thus, the issue of whether fibrosis‐linked microRNAs are secreted from the cardiac tissue has yet to be resolved. In this study, corresponding microRNAs in blood and myocardial tissue were examined simultaneously, and the absence of any correlation between them was clearly demonstrated.

Preliminary studies showed that ECM fibrosis was associated with increased expression of pro‐fibrotic microRNAs, including miR‐21 and miR‐208a and decreased expression of anti‐fibrotic microRNAs, including miR‐29, miR‐30 and miR‐133.^2^, ^3^, ^4^ In accordance with these findings, we observed that myocardial miR‐29 was up‐regulated in our DCM cohort in comparison with CABG patients, whereas myocardial miR‐26 and miR‐133a were down‐regulated in DCM. Thus, the aetiology of HF seems to be crucial with respect to molecular cardiac pathology, including the distribution of microRNAs. We have previously reported relatively strong correlations between circulating miR‐26 and miR‐30 with CVF (*r* = .48, *P* < .01 and *r* = .72, *P* < .001, respectively).^11^ Surprisingly, the corresponding myocardial microRNAs did not have any relationship with ECM fibrosis. In a predominantly inflammatory DCM cohort, Besler et al^13^ found that increased myocardial expression of miR‐133 was weakly associated with fibrosis. The aetiology seems to be of paramount importance as we did not observe any inflammatory infiltrations on biopsy, whereas Besler et al studied solely inflammatory DCM.

Bio‐SHiFT investigators showed that of 7 circulating microRNAs previously linked to HF, only miR‐22 was associated with the end‐point in a large cohort of unselected HF patients.^14^ Our study reveals that circulating microRNAs we selected to examine have no role in the prognosis in DCM. In a recent study, Besler et al^13^ reported that myocardial miR‐133a expression was related to CV outcome in inflammatory DCM. Our findings extend this observation to the non‐inflammatory DCM population.

### 4.1 Study limitations

The number of patients in the control cohort is small, which was as a result of a slow recruitment rate. Only selected microRNAs can be measured with qPCR. Although next‐generation sequencing enables the analysis of virtually all microRNAs, we chose to use qPCR as our aim was to verify the role of the most “promising” microRNAs in relation to fibrosis.

## 5 CONCLUSIONS

The lack of correlation between corresponding circulating and myocardial microRNAs calls into question their cellular source. Based on the expression of a selection of circulating microRNAs (miR‐21, miR‐26, miR‐29, miR‐30 and miR‐133a), no prediction can be made on the expression of their myocardial counterparts. No associations were found between myocardial microRNAs and fibrosis. Only myocardial miR‐133a was found to be an independent predictor of prognosis. Although preliminary, this study sheds new light on the role of microRNAs in DCM pathology and warrants further exploration.

## CONFLICT OF INTEREST

The authors confirm that there are no conflicts of interest.
